# Optimization of Casting Process Parameters for Solidification Structures in Complex Superalloy Castings

**DOI:** 10.3390/ma18174205

**Published:** 2025-09-08

**Authors:** Shaoli Han, Heli Luo, Shangping Li, Guangwei Han

**Affiliations:** 1 High-Temperature Materials Research Institute, Central Iron and Steel Research Institute, Beijing 100081, China; 13801373343@163.com (H.L.); 13521299753@163.com (S.L.); zxe221@163.com (G.H.); 2 Beijing CISRI-GAONA Materials & Technology Co., Ltd., Beijing 100081, China

**Keywords:** casting the grain structure, filling flow field, pouring temperature, shell temperature, cooling rate

## Abstract

This study aimed to optimize the grain structure of complex thin-walled nickel-based superalloy castings by investigating the influence of key casting parameters using both cellular automaton–finite element (CAFE) simulations and experimental validation. The main problem addressed was the inhomogeneous grain morphology arising from complex mold geometries and uneven thermal conditions during investment casting. The solidification process was simulated using the ProCAST software, incorporating the CAFE method to model temperature fields and grain growth dynamics. The results revealed that the molten metal flow pattern during mold filling significantly affected the local temperature field and subsequent grain formation. Specifically, simultaneous bidirectional filling minimized thermal gradients and suppressed coarse columnar grain formation, promoting finer, more uniform equiaxed grains. Lowering the pouring temperature (to 1430 °C) in combination with reduced shell temperature (600–800 °C) enhanced nucleation and improved grain uniformity in thin-walled regions. Higher cooling rates also refined the grain structure by increasing undercooling and limiting grain growth. Experimental castings confirmed these simulation outcomes, demonstrating that the proposed optimization strategies can significantly improve grain homogeneity in critical structural areas. These findings provide a practical approach for controlling microstructure in large, intricate superalloy components through targeted process parameter tuning.

## 1. Introduction

Nickel-based superalloys solidified in equiaxed grains are widely acknowledged for their cost-effective manufacturability and superior mechanical performance under medium to low temperature conditions, making them extensively utilized in the aerospace and aviation sectors. For cast components operating at elevated temperatures within similar strength regimes, enhancing fatigue resistance and extending service life typically necessitate the development of a fine and uniform grain structure throughout the casting [1,2]. Nickel-based superalloys are a class of high-performance alloys primarily composed of nickel, along with elements such as chromium, cobalt, aluminum, and titanium. These alloys exhibit exceptionally high-temperature strength, resistance to thermal creep deformation, and excellent oxidation and corrosion resistance, which makes them particularly appropriate for turbine engines and other aerospace applications.

In recent years, efforts to reduce fuel consumption and improve aircraft engine efficiency have driven annual increases in engine thrust-to-weight ratios, resulting in higher operating temperatures. Aircraft engines, which are subjected to high temperatures and rapid rotational sequences, have experienced continuous advancements in materials and significant structural optimizations across generations, leading to increasingly intricate designs [3,4]. The casing castings, which exemplify these complex structures, feature inner and outer ring configurations. These rings are connected by eight ribs containing internal cavity structures, with thick-walled mounting flanges positioned at the front and rear to enable integration with other engine components. The overall structure resembles a cylindrical shell with multiple interconnected thin-walled and thick-walled regions, including radial support ribs that contribute to significant spatial complexity. The ribs form narrow internal passages that are challenging to fill uniformly during casting, while the mounting flanges act as thermal masses that exacerbate solidification heterogeneity. These design features collectively result in steep thermal gradients and directional heat flows, making the control of grain morphology particularly difficult. Serving as the primary load-bearing structure in an aircraft engine, the turbine load-bearing casing supports all thrust and vibration loads, thereby playing a pivotal role in the engine’s performance and durability. The stringent service requirements imposed on the casing necessitate the absence of cracks, porosities, and other metallurgical defects, with the solidification microstructure being equally critical. Variations in cross-sectional thickness and the influence of the flow field lead to differential cooling rates across the wall sections, resulting in pronounced heterogeneity in the grain structure of the casing. As a result, the mechanical performance of the casing in sample tests often falls short of expectations [5].

With increasing design complexity and a heightened focus on structural weight reduction, the production of defect-free, high-quality castings for large, complex, and thin-walled components has become a major technological challenge. Achieving uniform grain structure across the various structural regions of casing castings remains a critical barrier to meeting the demands of high-performance applications [6,7]. In this context, “casing castings” refer to large, load-bearing structural components of turbine engines—typically produced by investment or sand casting rather than high-pressure die casting, due to their size, complexity, and the use of high-temperature nickel-based superalloys. The solidification process itself is inherently complex, encompassing transitions from high-temperature melt to a liquid-solid biphasic region and eventually to a fully solid phase. Throughout these stages, the alloy’s thermophysical properties undergo substantial transformations. The traditional research on the solidification process of castings relies primarily on experimental observations and empirical summaries. However, the high cost of experiments, especially for large and complex castings, poses challenges in real-time observations of grain structure changes during solidification, parameter control, and reproducibility. In contrast, computer simulations can precisely and quantitatively elucidate various phenomena and their evolutionary patterns during alloy solidification. They have become increasingly important in the process optimization of casting development [8,9,10]. Computer simulations can be used to conduct extensive virtual experiments at a lower cost and visualize microscale changes. Common grain structure simulation methods include first-principle calculations, molecular dynamics, the Monte Carlo method, and the phase-field method, which can simulate larger-scale grain growth but face difficulties in handling complex geometries. The finite element method (FEM) is well-suited for calculating macroscopic temperature and stress fields; however, it presents challenges in directly simulating grain structure evolution [11,12,13,14,15,16,17]. The CA-FD/FE coupling model integrates the microscopic simulation capabilities of cellular automata (CA) with the macroscopic computational strengths of finite difference (FD) and finite element (FE) methods. This hybrid approach enables simultaneous consideration of macroscopic heat conduction and microscopic grain growth, providing high computational efficiency and suitability for large-scale castings with complex geometries [18,19]. Nonetheless, existing literature indicates limited progress in simulating grain structures in castings with intricate geometries, primarily due to the absence of advanced algorithms capable of handling complex morphologies and the multifaceted physical interactions occurring during solidification. Accordingly, this study aims to enhance the grain growth algorithm within the CA-FD/FE framework to more accurately capture the grain evolution behavior in castings with complex geometrical features.

This study details the modeling of representative structural elements of the turbine casing through an integrated methodology that couples cellular automata with finite element analysis to simulate and predict grain structure evolution during solidification. By calibrating parameters, such as bulk nucleation density and undercooling levels, the study targets precise control of grain morphology. With a focus on grain structure regulation, the effects of various casting techniques on grain distribution across different regions of the casing are systematically assessed. This methodology facilitates the optimization of casting parameters and the development of control strategies to mitigate undesirable microstructures—including localized columnar grains, coarse and fine equiaxed grains, and coarse dendritic formations—ultimately providing a robust framework for controlling solidification structures to improve the operational reliability and performance of the final turbine casing.

## 2. Materials and Methods

### 2.1. Design of the Structural Features of Casings

**Figure 1:**
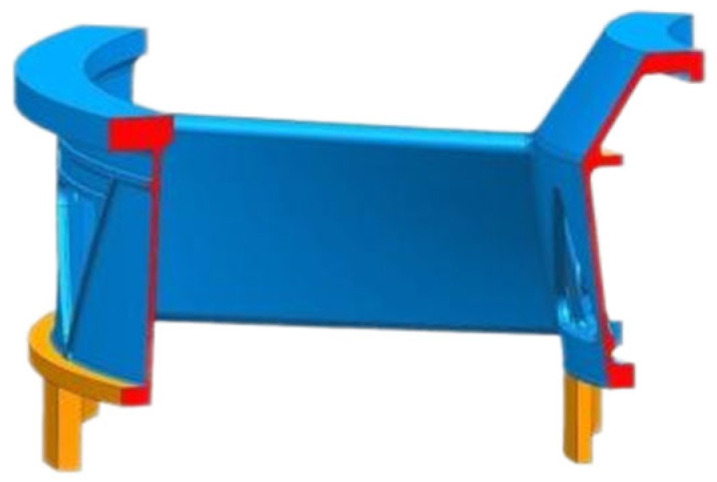
1/8 structural features of the casing.

**Figure 2:**
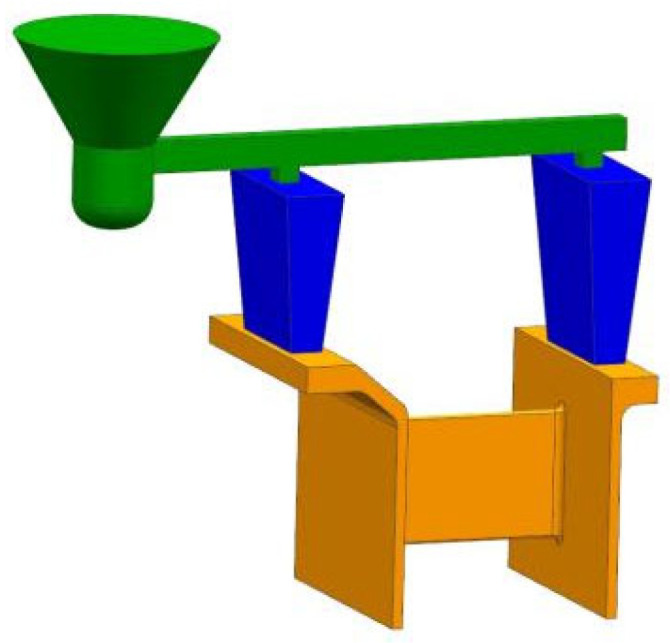
Structural features and gating system design.

As illustrated in Figure 1, the 1/8 casing structural assembly comprises several primary components: an outer ring, an inner ring, a support plate, and upper and lower mounting structures for both the inner and outer rings. Based on the structural characteristics of the casing shown in Figure 2, a representative structural element has been designed to capture the defining features of the casing. This component incorporates a top-gating system to facilitate sequential solidification of the casting. The characteristic zones of the casing are defined as follows:

(1) Left and right thin-walled plates (2 mm thick): These plates represent the inner and outer ring structures.
(2) Central connecting plate (1 mm thick): This plate represents the support plate linking the inner and outer rings.
(3) Mounting edge (5 mm thick): Incorporated at one end of the left and right thin-walled plates.

This representative structural component encapsulates all typical features of the casing, thereby enabling grain structure evolution studies using this model to accurately reflect the solidification behavior of the entire casing assembly. Its design facilitates targeted investigation into microstructural evolution in critical regions of the casing. By incorporating essential structural elements—such as thin-walled sections, connecting plates, and mounting edges—this model offers a comprehensive platform for analyzing solidification dynamics and the resulting grain structures across diverse features of the casing. As shown in Figure 1, the representative 1/8 casing component includes clearly defined structural parts: the inner ring (left vertical curved plate), outer ring (right vertical curved plate), central support plate (horizontal wall connecting the inner and outer rings), and the mounting edges (bottom extensions of the ring walls that provide attachment surfaces). The section view reveals the thickness variation across the different zones, highlighting the transition from thin-walled to thick-walled regions that influence heat extraction and solidification behavior.

In Figure 2, the complete casting system is illustrated, comprising the pouring cup (green funnel-shaped top element), sprue and runner system (green horizontal channel distributing molten metal), gating cones (blue elements regulating flow), and risers (blue conical reservoirs located above the casting mold cavity). The riser design ensures directional solidification by supplying liquid metal during the shrinkage in solidification. The risers are symmetrically positioned above the mounting zones to compensate for volume loss during cooling in these thicker regions. The mold cavity (orange zone) represents the actual casting geometry that includes the outer ring, inner ring, and connecting plate. This gating and riser configuration promotes a stable filling pattern, reduces turbulence, and minimizes the risk of defects, such as shrinkage porosity or cold shuts.

The purpose of simulating the grain structure after solidification is to predict the microstructural characteristics that directly influence the mechanical properties and performance of nickel-based superalloy castings. Grain size, morphology, and orientation significantly affect the creep resistance, fatigue strength, and thermal stability of these alloys, especially in complex geometries, such as turbine casings. By analyzing the solidification grain structure in representative structural components, this study aims to evaluate how various casting parameters influence grain evolution, identify potential defect-prone regions, and provide guidance for optimizing process conditions to ensure desirable microstructural outcomes and improve the overall quality of the final casting.

To simulate the grain structure evolution during solidification, a cellular automaton–finite element (CAFE) coupling model was employed. The finite element component was used to solve the transient heat transfer equation and establish the temperature field, while the cellular automaton method captured the nucleation and grain growth behavior. Progressive nucleation was incorporated by assigning spatially and temporally dependent nucleation probabilities to cells as a function of local undercooling. This approach allows continuous grain formation during solidification rather than instantaneous nucleation, better reflecting the physical casting process. The grain growth was governed by an anisotropic kinetics model, where the growth velocity was determined by local thermal gradients and the crystallographic orientation of neighboring grains. The CAFE model was implemented on a meshed domain representing the representative structural component of the casing, enabling a detailed investigation of solidification dynamics across distinct geometrical regions. This modeling framework supports the quantitative analysis of grain size distribution, morphology, and orientation under various thermal and structural conditions.

The dendrite tip growth kinetics were modeled using the Kurz–Giovanola–Trivedi (KGT) equation, which relates the dendrite tip velocity *V* to the local undercooling *ΔT*. The growth velocity was calculated using the expression:

$$V={\mu \cdot (\Delta T)}^{n}$$

where *μ* is the kinetic coefficient and *n* is the growth exponent, both of which were calibrated based on thermophysical properties of the Ni-based superalloy. This relation captures the dependence of dendritic growth rate on local thermal conditions and enables accurate prediction of grain morphology evolution during solidification. The anisotropy of the dendritic growth was also considered to account for crystallographic orientation effects on tip velocity.

To simulate the solidification process and microstructural evolution, a CAFE coupling framework was implemented, following the principles outlined previously [20]. In this hybrid model, the finite element method (FEM) was used to solve the transient heat conduction equation and generate the macroscopic temperature field throughout the casting domain. The CA component, overlaid on the FE mesh, resolved grain nucleation and growth by applying local transformation rules at the mesoscopic scale. Nucleation occurred progressively in the undercooled liquid and was statistically governed by a Gaussian probability distribution, while dendrite growth was modeled using anisotropic kinetics based on local thermal gradients and the KGT growth law. Grain competition, impingement, and crystallographic orientation selection were captured by orientation-dependent growth velocities within the CA grid. The CAFE approach enables a direct link between thermal conditions and grain structure evolution, making it particularly effective for complex casting geometries, such as the thin-walled casing examined in this study.

To characterize nucleation kinetics during solidification, a Gaussian (normal) probability distribution was employed to describe the statistical variation of nucleation temperatures within the undercooled melt. The nucleation density was defined as a function of local undercooling, with the nucleation probability peaking near the mean nucleation undercooling and tapering off symmetrically, as governed by the standard deviation. This approach enabled the modeling of progressive nucleation behavior throughout the domain, accounting for spatial and temporal thermal gradients during the casting process.

### 2.2. Numerical Simulation

**Figure 3:**
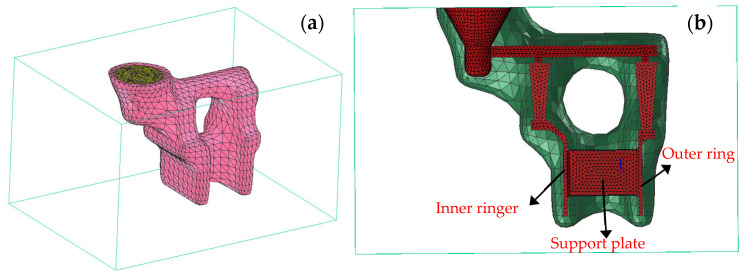
Simulation model and grid division. (**a**) simulation model; (**b**) cross-section of the simulation model.

This study uses ProCAST software to conduct numerical simulations of the solidification process in precision casting for the feature structural components of casings. Specifically, ProCAST 2022.0 (ESI Group, Bagneux, France) was employed, which provides comprehensive functionality for casting process simulation, including modules for heat transfer, fluid flow, phase transformation, and microstructure evolution. The simulation in this study utilized the Advanced Thermal and Microstructure Modules, enabling coupled heat transfer analysis and grain growth modeling via the CAFE approach. The mesh generation and boundary condition definitions were performed within the ProCAST Integrated Environment (PreCAST), while the simulations were solved using the solver engine CastSolver. Post-processing and grain structure visualization were carried out in Visual-CAST. Figure 3 presents the finite element mesh of the simulation model, with a mesh element size of 2 mm. The FE model was discretized using tetrahedral elements, with a global element size of 2 mm to balance computational cost and simulation accuracy. The complete mesh of the representative structural component consisted of approximately 160,243 elements and 36,907 nodal points, ensuring adequate spatial resolution in thin-walled regions and mounting structures where steep thermal gradients and rapid solidification are expected. Local mesh refinement was applied around the top-gating area and at the interfaces between different structural zones to accurately capture heat transfer and solidification front evolution. This mesh configuration provided sufficient fidelity to support both the macro-scale thermal field calculations and the meso-scale grain structure modeling within the CAFE framework. The experimental material for the casing components is a high-strength nickel-based alloy. The main chemical composition of the nickel-based superalloy (wt. %) was as follows: 9–11% Co, 2–4% W, 2–4% Mo, 5–7% Al, 5–7% Ti, 10–14% Cr, 0.05–0.15% B, and Ni balance. The shell, made of mullite sand, has a thickness of 8 mm. An external sandbox surrounds the shell, and silicon dioxide is used as the sand material. The thermal properties of the materials are summarized in Table 1 and are incorporated into the ProCAST material database. Based on empirical data, the interfacial heat transfer coefficient between the casting and the shell is sourced from ProCAST’s database for the In738-Mullite system. The heat transfer coefficient between the shell and the sandbox is set at 200 W/m^2^·K, with the sandbox temperature maintained at 600 °C. The heat transfer coefficient between the sandbox and the external furnace is specified as 20 W/m^2^·K, with the ambient furnace temperature held at 50 °C.

The simulation incorporates various casting process parameters, including three filling techniques regulated by ingate size and pouring speed: inside-out filling, simultaneous side filling, and outside-in filling. Pouring temperatures are set at 1400 °C, 1430 °C, and 1460 °C, while shell temperatures are adjusted to 600 °C, 800 °C, and 1000 °C. Regarding the cooling method at a shell temperature of 800 °C, two approaches are examined: one involves placing the shell directly into the furnace environment, resulting in a cooling rate of 23.7 °C/s at the support plate region; the other involves wrapping the shell in insulating cotton prior to furnace placement, reducing the cooling rate to 18.5 °C/s in the same region. Under these varying process conditions, the CAFE grain growth coupling method is employed to simulate the solidification grain structure of the representative structural components of the casing.

To promote a clearer understanding of the internal solidification behavior and sampling locations, a cross-sectional view of the simulation model has been added to Figure 3. This sectional representation highlights the characteristic zones, such as the inner ring, outer ring, and support plate, from which metallographic samples were extracted and analyzed, enabling direct comparison between simulated and experimentally observed grain structures.

The CAFE approach is an innovative computational framework that combines macrolevel heat transfer analysis with microlevel grain growth modeling, and it is widely used for predicting solidification structures via various casting methods. Among these, simulating the temperature field is a crucial foundation for grain structure modeling, as described by the governing equation [15] in Equation (1):

(1) $${\rho c}_{p}\frac{\partial T}{\partial t}=\lambda \cdot \nabla^{2}T+\rho \Delta H\frac{\partial f_{s}}{\partial t}+Q_{R}$$

where T represents temperature, t denotes time, ρ signifies density, Cp represents specific heat capacity, ΔH refers to latent heat, λ indicates thermal conductivity, fs represents the solid fraction, and Q_R_ accounts for the thermal exchange between the casting and its surroundings. In this study, the density term ρ in Equation (1) refers to the nickel-based superalloy used in the casting simulation and ranges from 6530 to 7770 kg/m^3^, as presented in Table 1. This range accounts for the temperature-dependent variation in density during the solidification process.

Grain structure modeling utilizes an enhanced cellular automaton (CA) technique [24]. Grain nucleation during solidification involves both homogeneous and heterogeneous mechanisms, with heterogeneous nucleation being the dominant mode. It is assumed that nucleation is initiated once a critical undercooling, ΔT, is reached, thereby activating a corresponding number of nucleation sites. As the degree of undercooling increases, the nucleation density correspondingly rises, as characterized by Equation (2):

(2) $$\frac{dn}{d\left(\Delta T\prime \right)}=\frac{n_{max}}{\sqrt{2\pi }∆T_{\sigma }}exp\left[-\frac{1}{2}{\left(\frac{∆T\prime -∆T}{∆T_{\sigma }}\right)}^{2}\right]$$

The nucleation density under a certain degree of supercooling can be calculated via Equation (3):

(3) $$n\left(∆T\right)=\int_{0}^{∆T}\frac{d_{n}}{d\left(∆T\prime \right)}d\left(∆T\prime \right)$$

Grain growth is computed using the KGT dendrite growth model, which determines the growth rate of the dendritic tip. The simulation outcomes effectively capture the phenomena of columnar crystal competition and centrally located equiaxed crystal growth, as described by Equation (4) [25]:

(4) $$v\left(∆T\right)=\alpha_{2}∆T^{2}+\alpha_{3}∆T^{3}$$

where α_2_ and α_3_ are dendritic growth kinetic parameters specific to the alloy system. For the nickel-based superalloy used in this study, α_2_ = 6.3 × 10^−7^ m^2^/s^2^ and α_3_ = 3.33 × 10^−6^ m^2^/s^2^. In addition, the CAFE grain growth models included ΔT_s,max_ = 15 K, ΔT_s_,σ = 1.5 K, n_s,max_ = 2.5 × 10^6^ m^–3^, ΔT_v,max_ = 15 K, ΔT_v,σ_ = 2.5 K, n_v, max_ = 3.6 × 10^7^ m^–3^ (the facial kernel parameters are s, and the body core parameters are v).

#### Shell Material Selection and Mold Design

The use of mullite sand for shell fabrication is based on its superior thermal stability, low thermal expansion coefficient, and high refractoriness, making it well-suited for high-temperature applications, such as precision casting of nickel-based superalloys. Mullite (3Al_2_O_3_·2SiO_2_) exhibits excellent resistance to thermal shock and chemical attack, ensuring dimensional stability and structural integrity of the shell during metal pouring and solidification. These characteristics help prevent shell cracking and minimize casting defects associated with mold-metal reactions. The shell molding process involves multiple slurry and stucco layers using mullite as both the filler and stucco material. After each coating and drying step, the shell is built up to a final thickness of 8 mm to provide sufficient mechanical strength and thermal resistance.

Additionally, silica sand is used as the backfill material in the external sandbox to provide thermal insulation and support during casting. Although silica has a lower refractoriness and higher thermal expansion compared to mullite, it is economical and serves effectively as a secondary containment material that buffers heat loss and controls the cooling rate of the shell. The contrasting thermal conductivities of mullite (3.8–7.1 W/m·K) and silica (approximately 0.7 W/m·K) are accounted for in the simulation to accurately model heat transfer and solidification behavior.

### 2.3. Examination of the Grain Structure of the Casing

On the basis of the grain structure simulation results, a comparative analysis of the grain structure in the casing components under different process parameters was conducted. Metallographic samples were taken from selected areas of the outer ring, inner ring, and support plate of the casing components. These samples were chemically etched with H_2_O_2_:HCl = 1:1 (volume ratio), and their grain structure characteristics were subsequently examined with a Leica DM600M optical microscope (OM) (Wetzlar, Germany).

## 3. Results

This study employed the CAFE model to simulate the grain structure of complex thin-walled components. While the CAFE method offers significant advantages, it exhibits limitations in capturing intricate physical and metallurgical phenomena, particularly those related to constitutional undercooling resulting from solute diffusion during solidification. These limitations may contribute to deviations between simulated and actual grain structures observed in castings. Nonetheless, under consistent conditions, the parametric influence on grain structure derived from the simulations provides meaningful guidance for controlling grain morphology during the casting of complex thin-walled components. However, the current model does not explicitly account for constitutional undercooling effects caused by solute redistribution ahead of the solid–liquid interface. This phenomenon, which influences the stability of the solidification front and promotes equiaxed grain formation under certain conditions, is especially relevant in alloy systems with strong solute partitioning. The omission of these effects may lead to discrepancies in predicting the extent of columnar-to-equiaxed transitions and in accurately capturing grain refinement phenomena in solute-rich regions. Future improvements to the model should incorporate solute transport dynamics and their impact on constitutional undercooling to enhance predictive accuracy.

### 3.1. Effects of the Filling Flow Field on the Grain Structure

**Figure 4:**
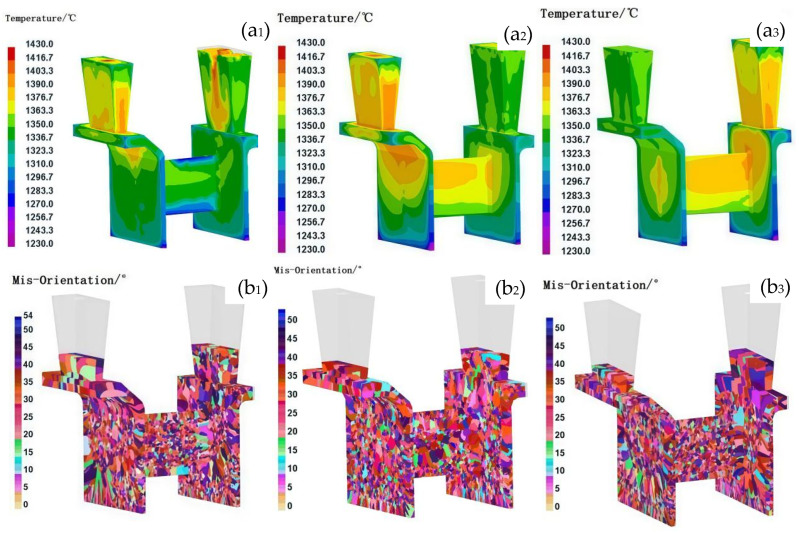
Comparison of the temperature field and grain structure of castings under different filling flow field conditions. (**a**) Temperature field, (**b**) grain structure; **1**, **2**, and **3** represent the filling flow fields from inside to outside, inside and outside at the same time, and from outside to inside, respectively.

During the casting process, the filling flow field modified the temperature distribution of the melt, leading to localized changes in the temperature gradient and consequently affecting the nucleation rates and grain growth direction. A non-uniform flow field could result in a significant temperature gradient, which may cause the formation of localized columnar grains. In contrast, a uniformly distributed filling flow field could promote the formation of homogeneous equiaxed grains. In this study, three distinct filling methods were applied to the casing feature components by modifying the inner gate dimensions and pouring velocity: interior-to-exterior filling, simultaneous interior and exterior filling, and exterior-to-interior filling. Figure 4 presents the solidification temperature fields and grain structure characteristics of the casing components under different filling flow field conditions. With identical casting parameters (pouring temperature: 1430 °C, shell temperature: 800 °C, and sandbox temperature: 600 °C), these filling strategies significantly influenced the temperature distribution during the initial stages of solidification. The most uniform temperature distribution was observed with simultaneous interior and exterior filling, whereas unidirectional filling led to a progressively decreasing temperature gradient along the direction of molten metal flow. As a result, three distinct grain structures emerged in the casing components: coarse columnar grains formed at the junction plate and L-shaped corners of the inner and outer rings under unidirectional filling, while fine equiaxed grains appeared at the center of the 1 mm-thick junction plate. In contrast, concurrent double-sided filling produced a more homogeneous grain structure, with no coarse columnar grains at the L-shaped corners, although some inconsistency remained in the uniformity of the grain structure at the center of the 1 mm-thick junction plate. These results demonstrate that the filling flow field directly influences the temperature distribution, which subsequently governs the resulting solidified grain structure. The following analysis explains the mechanisms behind grain structure formation under different filling conditions:

In unidirectional filling, the elevated initial solidification temperature in the junction plate region prevents non-uniform nucleation upon initial contact between the molten alloy and the shell, resulting in a uniform nucleation distribution. Under the condition of a non-uniform flow field, when the molten alloy first contacted the shell, the lower temperature of the shell promoted a higher degree of undercooling, which facilitated nucleation, leading to a greater number of nuclei. As the filling process continued, the shell temperature gradually increased, resulting in a decreased degree of undercooling and consequently a reduction in the number of nuclei formed in the subsequent stages of filling. The inconsistency in nucleation between the early and later stages of filling can readily result in an uneven grain structure distribution. At the L-shaped corners of the junction plate, columnar grains formed perpendicular to the sidewall, aligning with the molten alloy’s cooling direction due to the presence of a high temperature gradient. Additionally, the pronounced temperature gradient along the inner and outer sidewalls under unidirectional filling contributed to the development of coarse columnar grains on both the inner and outer ring sidewalls.

In contrast, under conditions of a uniform filling flow field, the reduced filling distance minimized temperature variations across the shell during the filling process. This facilitated a more homogeneous distribution of nucleation sites throughout the casting. During concurrent filling from both the inner and outer peripheries, the support plate region—serving as the endpoint of molten alloy inflow—exhibited the lowest temperature. The rapid heat dissipation in this area increased the propensity for heterogeneous nucleation as the molten alloy made contact with the shell. Furthermore, the convergence of steel streams in the support plate region introduced turbulent flow, resulting in a non-uniform spatial distribution of nucleation sites. As the shell temperature in this region rose during filling, grains formed during subsequent solidification were more likely to develop into coarse grains. Conversely, in the L-shaped corner regions adjacent to the support plate, the concurrent inflow from both concentric rings reduced the local temperature gradient as the filling distance shortened, thereby mitigating the formation of excessively coarse columnar grains.

### 3.2. Influence of the Pouring/Shell Temperature on the Grain Structure

**Figure 5:**
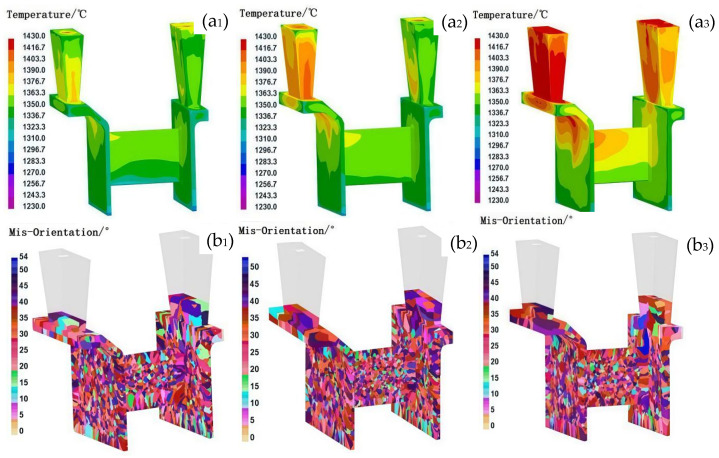
Comparison of grain organization under different pouring temperatures at the 1000 °C shell temperature. (**a**) Temperature field and (**b**) grain structure; **1**, **2**, and **3** represent pouring temperatures of 1400 °C, 1430 °C, and 1460 °C, respectively.

**Figure 6:**
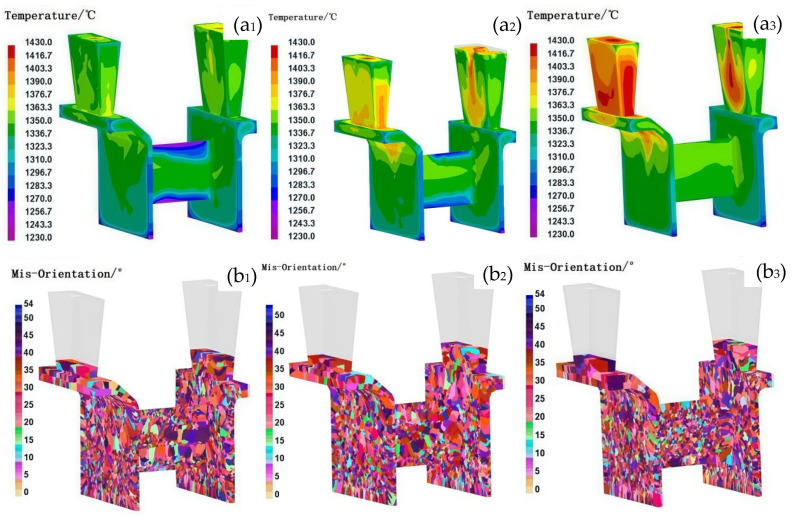
Comparison of grain organization under different pouring temperatures at 800 °C. (**a**) Temperature field and (**b**) grain structure; **1**, **2**, and **3** represent pouring temperatures of 1400 °C, 1430 °C, and 1460 °C, respectively.

**Figure 7:**
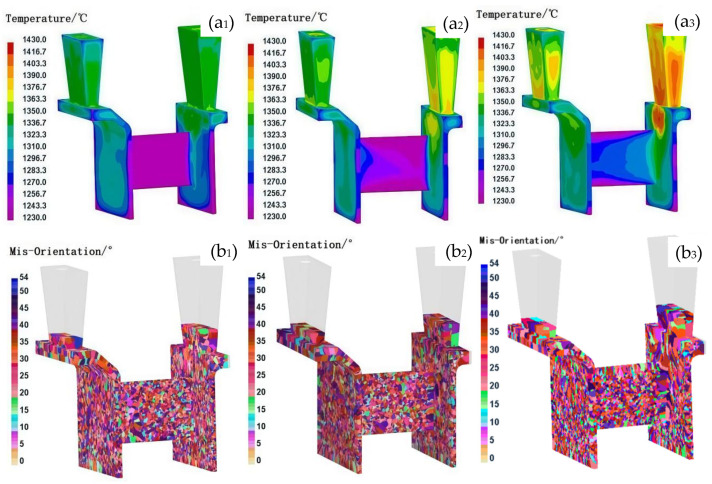
Comparison of grain organization under different pouring temperatures at the 600 °C shell temperature. (**a**) Temperature field and (**b**) grain structure; **1**, **2**, and **3** represent pouring temperatures of 1400 °C, 1430 °C, and 1460 °C, respectively.

In traditional investment casting processes, for casting with simple structures, the grain size increases with increasing pouring and shell temperatures. The uniformity of the grain structure improved with decreasing pouring temperature or increasing shell temperature. However, for thin-walled casts with complex structures, the impact of pouring and shell temperature on the grain structure varies with changes in the casting structure. On the basis of the consistent grain structure observed in the casing feature components during simultaneous filling on both sides, this study aimed to establish optimal casting parameters under controlled flow field conditions. Figure 5, Figure 6 and Figure 7 show the solidification temperature field and grain structure characteristics of the casing feature components under different pouring and shell temperature conditions. The comparison results indicate that the grain size increases as the pouring and shell temperatures increase. While both shell and pouring temperatures influence the thermal field and resulting grain structures, direct comparison of their relative effects is limited by the different step sizes used in this study (shell temperature: 200 °C; pouring temperature: 30 °C). Therefore, conclusions regarding which parameter has a greater influence should be made with caution. In terms of grain structure uniformity, a decrease in the pouring temperature improved the uniformity at lower shell temperatures, whereas at higher shell temperatures, increasing the pouring temperature led to a reduction in the number of elongated columnar grains but an increase in their size, thereby increasing grain structure heterogeneity.

The observed effects can be interpreted as follows: at a low shell temperature (e.g., 600 °C), the shell facilitates rapid cooling, which promotes the formation of numerous uniformly distributed nuclei, resulting in a fine and homogeneous grain structure. Additionally, under such conditions, the influence of pouring temperature on grain morphology is minimal. In thin-walled sections, the final grain structure is predominantly governed by the cooling rate, and the variation in cooling rates due to changes in pouring temperature becomes negligible under rapid cooling conditions [26]. However, as shell temperature increases, the impact of pouring temperature on grain structure becomes more pronounced. At a shell temperature of 800 °C, moderate thermal insulation leads to limited coarsening of grains in areas, such as the L-shaped corners and connection zones (Figure 6(b_1_)), although the effect is less remarkable than at 1000 °C. At a shell temperature of 1000 °C, the elevated thermal environment and prolonged solidification contribute to the formation of coarser grains in localized regions, particularly in the L-shaped corners and the central area of the connecting plate, as shown in Figure 5(b_1_). Elevating the pouring temperature under these conditions reduces the incidence of non-uniform nucleation, lowers the temperature gradient, and thereby improves the uniformity of the grain structure. This behavior contrasts with that observed in castings with thick cross-sections, where pouring temperature typically has a more uniform influence on grain refinement [27].

At a shell temperature of 1000 °C, the elevated thermal environment and extended solidification times can lead to partial remelting of previously solidified dendrites near the shell wall. This remelting increases the number of active nucleation sites [17,28], resulting in a more uniform grain structure compared to that formed at 800 °C, particularly in the thin-walled bracket sections with a thickness of 1 mm under identical pouring temperatures. Nonetheless, at such high shell temperatures, increasing the pouring temperature significantly affects grain size differentiation between thick- and thin-walled regions.

To achieve a uniform and fine-grained microstructure in complex thin-walled components, such as casing structures, several strategies can be employed. One effective approach involves lowering the pouring temperature to the range of 1400–1430 °C while maintaining a reduced shell temperature of 600 °C. This combination enhances nucleation and supercooling, thereby promoting the formation of numerous uniformly distributed nuclei under accelerated cooling conditions. However, this method may pose challenges in fully filling thin-walled sections due to reduced fluidity. Alternatively, employing higher pouring temperatures in conjunction with elevated shell temperatures may, under certain conditions, mitigate filling defects and promote acceptable grain structure uniformity in thin-walled regions. For instance, a shell temperature of 800 °C paired with a pouring temperature of 1460 °C, or a shell temperature of 1000 °C with a pouring temperature of 1430 °C, can reduce thermal gradients and suppress non-uniform nucleation. However, the resulting grain morphology tends to be coarser, and as observed in Figure 5 and Figure 6, such conditions may also lead to grain structure heterogeneity, particularly in regions such as L-shaped corners and central connecting plates. Therefore, while elevated pouring and shell temperatures may provide processability benefits, they must be optimized cautiously, as their combined use can also promote excessive grain growth, especially in thicker-walled regions of the casting.

### 3.3. Influence of the Cooling Rate on the Grain Microstructure

**Figure 8:**
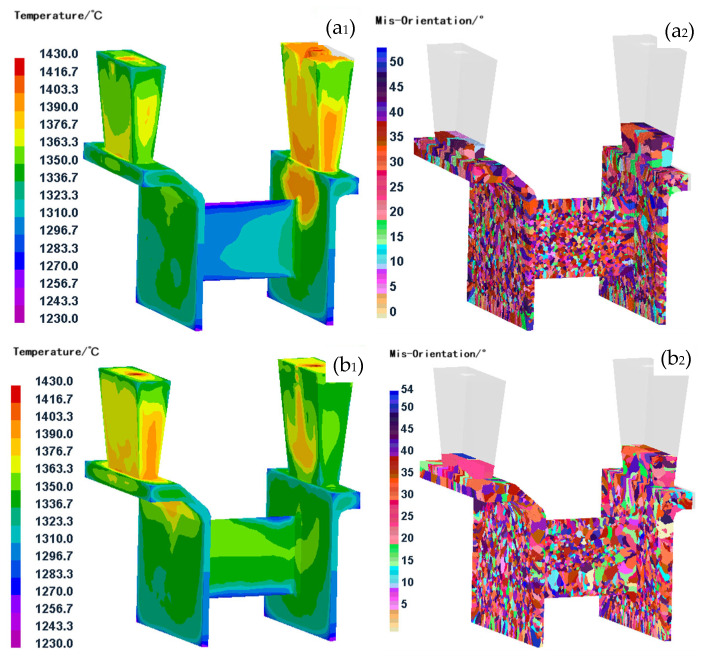
Comparison of temperature fields and grain structure under different cooling rates. (**a_1_**,**a_2_**) Rapid cooling rate condition; (**b_1_**,**b_2_**) slow cooling rate condition. (**a_1_**,**b_1_**) show the simulated temperature field distribution, while (**a_2_**,**b_2_**) depict the resulting grain structure visualized via grain misorientation.

In addition to the aforementioned influencing factors, the cooling rate of the casting also had a significant effect on the grain structure. Similar to the impact of the pouring temperature and shell temperature, for thin-walled casting with complex structures, the effect of the cooling rate on the grain structure was also influenced by the casting structure. Figure 8 presents a comparison of the grain structures in casing feature components under two distinct cooling rates, both conducted at a pouring temperature of 1430 °C and a shell temperature of 800 °C. The results indicate that in thin-walled structural regions, a higher cooling rate leads to a more refined and uniform grain structure compared to a lower cooling rate. This observation is supported by an analysis of nucleation site distribution and the temperature field. Under conditions of rapid cooling, nucleation sites are more uniformly distributed, particularly in the support plate region. The substantial degree of supercooling at the onset of solidification in this area promotes the generation of numerous nuclei. Additionally, the elevated cooling rate limits subsequent grain growth, thereby contributing to the formation of a fine-grained microstructure.

Conversely, under slower cooling conditions, higher overall temperatures, and continued molten alloy liquid filling influence the number of nucleation sites, leading to uneven nucleation distributions. Additionally, the greater driving force for grain growth under slow cooling conditions results in uneven grain distribution and excessive grain growth in thick-walled areas. In contrast to previous studies [17,18,19,20,21,22,23], this paper focused on investigating the influence of the pouring process parameters on the grain structure in different regions of complex thin-walled components, as well as the effect of the casting process parameters on the overall grain uniformity of the casting. The authors proposed innovative process control methods for determining the grain structure of a large, complex thin-walled superalloy components.

### 3.4. Comparison Experiment Analysis of the Solidification Grain Structure for Casings

**Figure 9:**
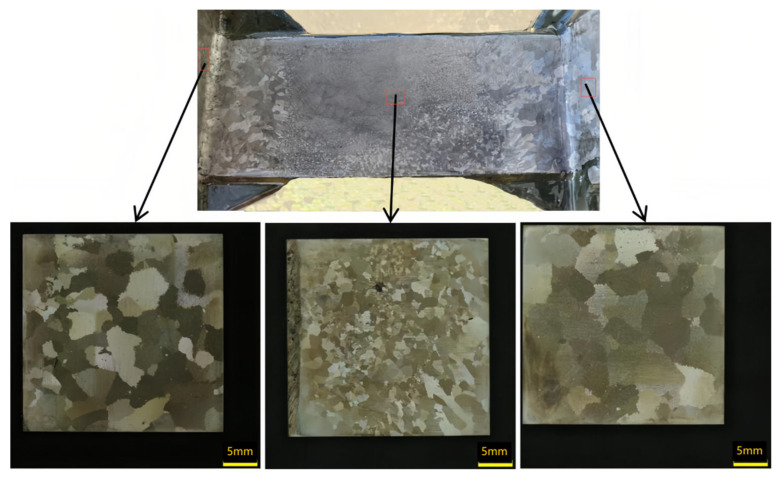
Casting condition 1 (pouring temperature of 1460 °C, shell temperature of 950 °C, and single-sided filling).

**Figure 10:**
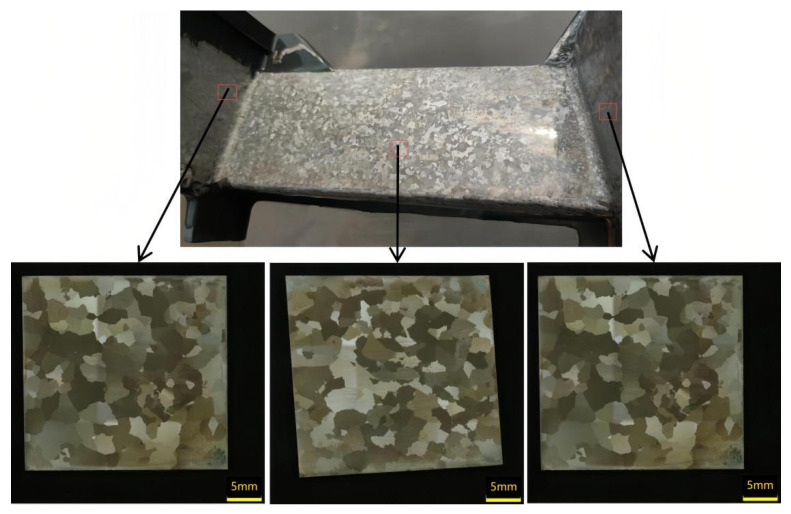
Casting condition 2 (pouring temperature of 1430 °C, shell temperature of 950 °C, and double-sided filling).

This study aims to validate the previously discussed simulation results and optimize casting process parameters for nickel-based superalloy casing components through practical casting experiments. The primary objective is to achieve an optimal balance between grain refinement and structural uniformity, while minimizing excessive grain growth, particularly in regions with variable wall thickness. Figure 9 and Figure 10 illustrate the grain structure characteristics observed in various regions of the casing components—specifically, the outer ring, inner ring, and support plate—under a constant mold temperature of 950 °C, with variations in process parameters, such as filling method and pouring temperature.

Figure 9 depicts the grain structure resulting from high-temperature, slow, unidirectional filling, consistent with simulation predictions. As predicted in the simulations (Section 3.1, Figure 4(b_1_,b_2_)), the combination of a 1460 °C pouring temperature and elevated shell temperature leads to grain coarsening in regions of prolonged solidification or delayed filling. The structure displays marked heterogeneity: the central region of the support plate exhibits a fine equiaxed grain structure, whereas coarse columnar grains are present at the L-shaped corners adjacent to the inner and outer rings. These localized regions of coarser grains were previously identified in the simulation results as zones where delayed filling and reduced cooling rates allow columnar grain growth to dominate, particularly under unidirectional flow conditions. In contrast, Figure 10 shows the outcome after modifying the casting parameters based on the conditions in Figure 9. The pouring speed is increased to facilitate simultaneous filling of the inner and outer rings, and the pouring temperature is reduced from 1460 °C to 1430 °C. Compared to Figure 9, the faster, concurrent filling strategy in Figure 10 significantly suppresses the formation of columnar grains at the L-shaped corners. Although earlier results indicated that higher pouring temperatures enhance grain uniformity at elevated shell temperatures (Section 3.1), in this specific case, the improvement in uniformity is primarily attributed to the simultaneous filling strategy rather than the reduction in pouring temperature alone. This indicates that the influence of the filling flow field on grain structure is more substantial than that of pouring temperature, especially in thin-walled regions where thermal and flow asymmetries dominate the local solidification dynamics. Although earlier results indicated that higher pouring temperatures enhance grain uniformity at elevated shell temperatures (Section 3.1), in this specific case, the improvement in uniformity is primarily attributed to the simultaneous filling strategy rather than the reduction in pouring temperature alone. Moreover, the lower pouring temperature, when combined with concurrent filling, results in finer grains in the inner and outer rings near the riser region, thereby enhancing the overall uniformity of the grain structure across the casing components.

Table 2 provides a statistical analysis of the grain size distribution across various regions of the casing components under the two different casting process conditions.

The microstructural features observed in Figure 9 reflect a remarkable directional solidification behavior. The slow and high-temperature unidirectional filling promoted thermal gradients along the casting flow direction, resulting in coarse columnar grains at the L-shaped corners near the outer and inner rings. These regions solidified more slowly due to prolonged heat retention, allowing grains to grow directionally along the temperature gradient. In contrast, the central support plate, subjected to more rapid cooling due to its geometric isolation and greater surface-area-to-volume ratio, exhibits a fine equiaxed grain structure formed via constitutional supercooling and spontaneous nucleation.

Figure 10 demonstrates a marked improvement in grain refinement and uniformity. The increased pouring speed led to a more homogeneous temperature field and reduced solidification time across the casting. This shift diminished the thermal gradient-driven columnar growth and facilitated equiaxed nucleation throughout the casting. Furthermore, the lower pouring temperature increased the degree of undercooling, enhancing nucleation rates and reducing grain size in both the inner and outer rings. As a result, the microstructure in Figure 10 exhibits a more homogeneous distribution of fine equiaxed grains, indicating a successful suppression of undesirable columnar grain formation and improved structural integrity of the final component.

### 3.5. Sensitivity Analysis of Key Simulation Parameters

To assess the robustness of the CAFE model predictions, a sensitivity analysis was conducted by varying key simulation parameters, including pouring temperature, shell temperature, and cooling rate, within realistic process limits. The results revealed that grain size and morphology were highly sensitive to shell temperature variations (±100 °C), which significantly altered the cooling rates and nucleation densities, particularly in thin-walled regions. Pouring temperature changes (±30 °C) exerted a moderate influence, predominantly affecting grain uniformity at elevated shell temperatures. Cooling rate adjustments, simulated by modifying boundary heat transfer coefficients, had a significant effect on the refinement and distribution of grains, especially near the support plate and L-shaped corner regions. These findings support the conclusion that shell temperature and cooling rate are dominant factors in determining grain morphology, highlighting the importance of carefully controlled thermal conditions in casting complex thin-walled components.

A limitation of this study is the restricted number of experimental observations conducted across the entire casting, as validation was primarily based on selected regions, such as the outer ring, inner ring, and support plate. Additional experimental data from more diverse and intricate casting regions would be necessary to further substantiate the simulation predictions and enhance the generalizability of the findings.

## 4. Conclusions

Through research on the optimization of casting process parameters for controlling the grain structure of large, complex, thin-walled superalloy components, the following three main conclusions have been obtained:

(1) The filling flow field plays a critical role in achieving uniform grain distribution within casing structural components. Concurrent filling from both the interior and exterior, as opposed to unilateral filling, facilitates a more consistent solidification temperature gradient across casing features, thereby reducing the occurrence of abnormally coarse columnar grains at L-shaped corners. However, the convergence of two molten alloy streams may introduce turbulence, which can result in irregular grain distribution and localized regions of coarser grains. Consequently, for the casting of large, complex thin-walled components, a multi-gate, rapid filling flow field control strategy should be employed to improve grain structure uniformity across various regions of the casting.
(2) Both pouring temperature and shell temperature positively correlate with grain size, with shell temperature exerting a more significant influence. For fine and homogeneous grain structures, a lower pouring temperature is advisable at low shell temperatures. Conversely, at elevated shell temperatures, a moderate increase in pouring temperature can enhance heterogeneous nucleation, resulting in slightly coarser yet more uniformly distributed grains. Compared to slow cooling, rapid cooling more effectively promotes uniform grain nucleation within the thin-walled regions of casting features.
(3) During the casting process of the nickel-based superalloy casing, the fiilling flow field control with simultaneous filling of the inner and outer rings, combined with the optimal casting parameters—a pouring temperature of 1430 °C and a shell temperature of 950 °C—results in a uniformly fine grain structure distribution, with grain sizes ranging between 2 and 4 mm. At the L-shaped corner of the support plate, no coarse columnar grain structures are observed; similarly, the central region of the support plate does not exhibit abnormally fine grain structures.

## Figures and Tables

**Table 1:** Thermal property parameters of the materials.

Material Parameters	Density/ρ (g/cm^3^)	Thermal Conductivity/Cp (W/m·K)	Solidification Temperature/T (°C)	Melting Temperature (°C)	Enthalpy/H (J/g)	Viscosity/µ (MPa·s)
The nickel-based superalloy	7.77–6.53	14.72–35.27	1246–1355	1320–1360 [21]	0.97–1153.57	7.33–3.23
Mold-Mullite	3.15	3.8–7.1	/	1840–1850 [22]	0.55–1.2	/
Sand box-Silica	1.52	0.7	/	1710 [23]	0.68–1.23	/

**Table 2:** Grain size distributions of the casing features under different casting conditions (mm).

Casting Condition	Outer Ring	Support Plate	Inner Ring
condition 1	3–7	1–4	3–7
condition 2	2–4	2–3	2–4

## Data Availability

The raw data supporting the conclusions of this article will be made available by the authors on request.
